# Influence of health-system change on treatment burden: a systematic review

**DOI:** 10.3399/BJGP.2022.0066

**Published:** 2022-10-18

**Authors:** Kate SH Matthews, Susannah C Rennoldson, Simon DS Fraser

**Affiliations:** Faculty of Medicine, University of Southampton, Southampton.; Faculty of Medicine, University of Southampton, Southampton.; Faculty of Medicine, University of Southampton, Southampton.

**Keywords:** long-term conditions, multimorbidity, primary care, systematic review, treatment burden

## Abstract

**Background:**

Treatment burden is a patient-centred concept describing the effort required of people to look after their health and the impact this has on their functioning and wellbeing. High treatment burden is more likely for people with multiple long-term conditions (LTCs). Validated treatment burden measures exist, but have not been widely used in practice or as research outcomes.

**Aim:**

To establish whether changes in organisation and delivery of health systems and services improve aspects contributing to treatment burden for people with multiple LTCs.

**Design and setting:**

Systematic review of randomised controlled trials (RCTs) investigating the impact of system-level interventions on at least one outcome relevant to previously defined treatment burden domains among adults with ≥2 LTCs.

**Method:**

The Embase, Ovid MEDLINE, and Web of Science electronic databases were searched for terms related to multimorbidity, system-level change, and treatment burden published between January 2010 and July 2021. Treatment burden domains were derived from validated measures and qualitative literature. Synthesis without meta-analysis (SWiM) methodology was used to synthesise results and study quality was assessed using the Cochrane risk-of-bias (version 2) tool.

**Results:**

The searches identified 1881 articles, 18 of which met the review inclusion criteria. Outcomes were grouped into seven domains. There was some evidence for the effect of system-level interventions on some domains, but the studies exhibited substantial heterogeneity, limiting the synthesis of results. Some concern over bias gave low confidence in study results.

**Conclusion:**

System-level interventions may affect some treatment burden domains. However, adoption of a standardised outcome set, incorporating validated treatment burden measures, and the development of standard definitions for care processes in future research would aid study comparability.

## INTRODUCTION

The number of people with multiple long-term conditions (LTCs) is increasing, reflecting ageing populations worldwide, which is challenging for healthcare systems and services operating with finite resources.^1^ Multimorbidity, often defined as the coexistence of ≥2 LTCs, is more common with older age, but exhibits earlier onset among people from lower socioeconomic groups.^2^ It is associated with several adverse health outcomes, including poor quality of life, reduced functional ability, and increased mortality.^3^ Coordination of care for patients with multiple LTCs can be challenging in health systems that are structured for individual disease management.^4^

Treatment burden describes the workload of health care for patients, including self-management and treatment, and the impact such demands have on wellbeing and functioning.^5^ High treatment burden can be detrimental to quality of life and health outcomes.^6^ Efforts to respond to high treatment burden may involve either increasing patients’ capacity to manage, or reducing the workload imposed on them.^7^ Uncoordinated care may lead to increased complexity for patients and contribute to health-service inefficiency and ineffectiveness.^5^

In the UK, recent changes to health policy indicate a movement towards collaborative, integrated care models to improve care for patients with multiple LTCs.^8^^,^^9^ Such system-level changes have the potential to reduce treatment burden by operationalising the principles of minimally disruptive medicine (MDM), focusing on outcomes that are important to patients, reducing workload, and increasing capacity.^10^

A recent systematic review^11^ explored the effectiveness of patient-level interventions in reducing treatment burden, and several studies reported positive outcomes. However, conclusions were limited due to study heterogeneity and the risk of bias. Even less is known about the impact of system-level change on patient experience, particularly treatment burden. Given the lack of a widespread adoption of treatment burden measurement in health care or research, the systematic review presented here aimed to explore the effects of system-level change on prespecified treatment burden domains derived from validated treatment burden measures among patients with multiple LTCs.^12^^,^^13^

## METHOD

### Data sources and searches

The review was conducted according to the Preferred Reporting Items for Systematic Reviews and Meta-analyses (PRISMA) and synthesis without meta-analysis (SWiM) guidelines (Supplementary Table S1). The SWiM criteria^14^ recommend a transparent, structured approach to synthesis by reporting how studies are grouped, any standardised metrics used, the synthesis method, how data are presented, a summary of findings, and limitations of the synthesis. The review is registered on the international prospective register of systematic reviews (PROSPERO — https://www.crd.york.ac.uk/PROSPERO/) ID number: CRD42021265188.^14^^,^^15^

**Table:** How this fits in

The nature and extent of treatment burden experienced by patients with multiple long-term conditions is influenced by the way in which health systems are organised and operate, but little research, to date, has explored the impact of system-level change on treatment burden. In this systematic review of randomised controlled trials involving a wide range of interventions that considered domains of treatment burden as outcomes, some evidence of an effect of interventions, particularly those operating at local organisation level, was found. However, there are significant gaps in the evidence base, particularly the need to include validated treatment burden measures as outcomes in trials, and a lack of studies investigating interventions aiming to mitigate the financial impact and administrative workload for patients and carers. Clinicians and managers of primary care organisations should consider the impact of service organisation on patient and carer treatment burden.

The search strategy was developed with a senior librarian, and searches were undertaken using Embase, Ovid MEDLINE, and Web of Science during July 2021. The International Research Community on Multimorbidity repository and the National Grey Literature Collection were hand-searched for grey literature. Further references were requested through author follow-up, and the snowballing of citations identified additional relevant papers. Search terms (Supplementary Box S1) were formulated under five domains identified from the research question, which were:

- multiple conditions;
- long-term nature of disease;
- system-level change in care delivery;
- outcome measures within previously identified domains of treatment burden; and
- the study design of randomised controlled trials (RCTs).

Directly measured, self-reported treatment burden could not be used as the sole outcome measure because of a lack of studies using validated treatment burden measures. As such, treatment burden domains were formulated a priori using validated tools, Multimorbidity Treatment Burden Questionnaire (MTBQ) and Patient Experience with Treatment and Self-management (PETS) measures, and themes from an extensive qualitative literature review of 110 studies of patient capacity and constraints in the experience of chronic disease.^12^^,^^13^^,^^16^ Medical appointment load and medical expenses were included in both the PETS measure and the qualitative literature, and were, therefore, included as important domains a priori.^12^^,^^13^

The MTBQ was chosen because it is a 10-item measure validated in the UK, demonstrating good reliability as a corresponding measure of quality of life and patient-centred care.^13^ The items cover medication number, medication adherence, collecting prescriptions, monitoring health, arranging appointments, seeing multiple health professionals, attending appointments, disease knowledge, lifestyle changes, and help from family and friends. The MTBQ is limited by its lack of inclusion of financial burden, an important consideration, particularly in healthcare systems where treatment is not free at the point of use.^5^

The PETS, which was validated in English in the US, was chosen because of its comprehensive nature, covering 78 items over 15 content domains, and its wide use in multimorbidity domains.^12^ These domains include medical information and adherence, medical appointments, monitoring health, interpersonal challenges, medical and healthcare expenses, difficulty with healthcare services, role and social activity limitations, and physical and mental exhaustion.

Using a key qualitative study synthesising 110 reports of patient capacity and constraints in their experience of chronic disease, additional domains were formulated from themes in the review in order to include further relevant studies.^16^ These corresponded to areas also covered in the validated tools;^12^^,^^13^^,^^16^ as an example, health-related quality of life (HRQoL) was identified to be relevant within the domains of treatment burden in the qualitative study.

### Study selection

Studies were eligible for inclusion if they were in English and conducted in a population of adults:

- with multiple LTCs;
- when an intervention that could be defined as ‘system-level’ was applied; and
- when an outcome (primary or secondary) represented at least one of the treatment burden domains.

Adults were defined as those aged ≥18 years. Multiple LTCs was defined as a diagnosis of ≥2 LTCs, confirmed by the International Classification of Diseases (10^th^ version) codes for chronic conditions (https://icd10cmtool.cdc.gov/?fy=FY2022), or ≥5 long-term drug prescriptions, considered to reflect patients with multiple LTCs.

Eligible system-level interventions were identified through a definition adapted from a Cochrane review^17^ of complex interventions to improve outcomes in patients with multimorbidity in primary care settings. System-level was considered to represent three levels of change:

- clinician-level changes in care provision (structured management plans, scheduled follow-ups);
- changes in local organisational structure (multidisciplinary team care, collaborative care); and
- higher-level changes in care models (integrated care systems).^18^^,^^19^

The comparator was as defined in the included studies. Treatment burden domains, as defined above, were used as inclusion criteria for the outcomes, as an alternative to overall treatment burden measures.^12^^,^^13^^,^^16^

Two authors independently screened titles and abstracts, and applied inclusion/exclusion criteria. Disagreements were resolved by a third reviewer.

### Quality assessment

The quality assessment of each identified study was carried out independently by the two authors who had screened the titles and abstracts, and done using the algorithm-guided electronic Cochrane risk of bias 2 (RoB2) tool for RCTs.^20^ Disagreements were resolved by the third author *.*

### Data synthesis

Because of heterogeneity of study populations, interventions, comparators, and outcomes, meta-analysis was not considered possible. This review used the SWiM framework^14^ to synthesise results from included studies. Data from included studies were extracted into a standardised table, and studies were grouped according to outcomes categorised by treatment burden domains; this was considered the most transparent way to report the heterogeneous findings. Outcome data were summarised for each study using two domains chosen for prioritisation (impact on HRQoL and functional status), because of the higher proportion of studies with primary outcomes measured with these domains, as recommended by the SWiM criteria.

For those outcomes where synthesis was possible, the Grading of Recommendations, Assessment, Development and Evaluation (GRADE) approach^21^ was used to critically appraise the synthesised results and establish the confidence for the certainty of them, thereby guiding interpretation. However, because of study heterogeneity, and in view of the perceived risk of drawing misleading conclusions through SWiM-recommended methods (summary effect sizes, *P*-value combination, vote counting), data were not synthesised further. An evidence map was constructed to show gaps in the evidence base, with studies mapped by outcomes to previously identified domains and inclusion of additional pre-identified domains to show the scope of areas with limited research.

## RESULTS

### Included studies

**Figure 1.**
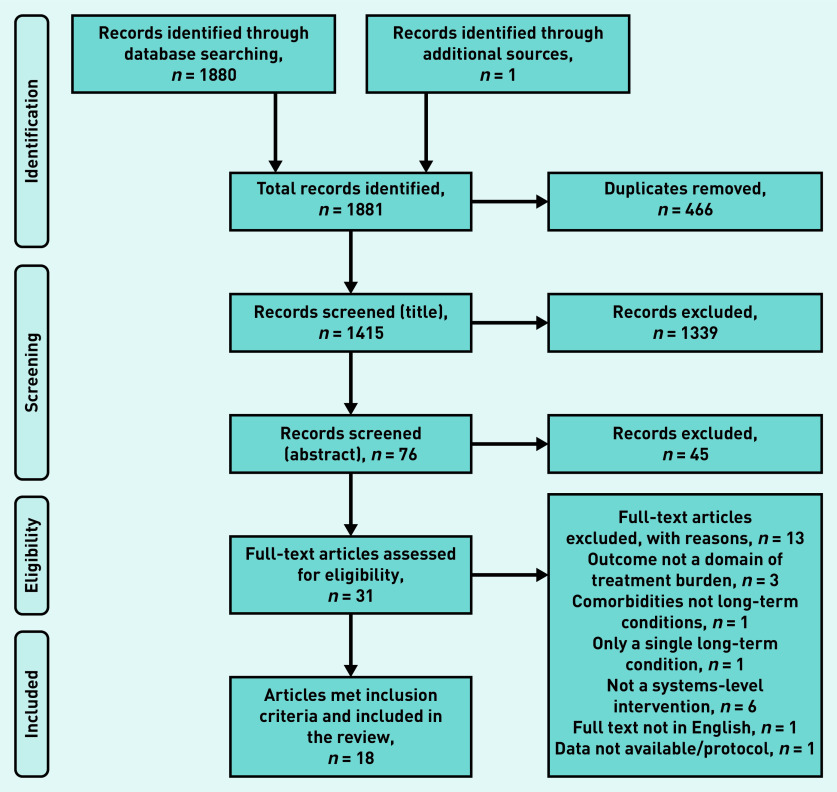
*PRISMA flowchart showing study selection process.*

In total, 1881 studies were identified from database searches, grey literature, and reference and author follow-up. Of these, 466 were duplicates and so were removed, leaving 1415 to be screened by title and abstract; 1384 studies were excluded for not meeting the inclusion criteria. Of the remaining 31 full texts that were assessed, 18 studies^22^^–^39 met the inclusion criteria (Figure 1).

### Study characteristics

Supplementary Table S2 summarises the characteristics of the included studies. There was considerable heterogeneity, which limited the potential for synthesis. The majority of studies were conducted in high-income countries with well-developed healthcare system structures. All studies were unblinded apart from one,^38^ which was able to single-blind participants to intervention receipt.

There was a spectrum of interventions across studies, from individual clinician-level to higher-level, cross-organisational change (Table 1). Five studies^25^^,^^27^^,^^28^^,^^31^^,^^37^ implemented interventions at clinician level. At local organisation level, three studies had a multidisciplinary approach to patient care,^34^^,^^37^^,^^38^ and nine used an intervention that could be considered collaborative care.^22^^–^24^,^^26^^,^^29^^,^^30^^,^^32^^,^^35^^,^^36^ One study^33^ used a collaborative care approach across multiple providers, which was considered a higher-level care model change.

**Table 1.** Intervention level and treatment burden domains of studies included in the review

**Study**	**Country**	**Participants, *n***	**Intervention level^a^**	**Treatment burden domains covered by outcomes**
Coventry *et al* (2015)^22^	UK	387	Local organisation	HRQoL, functional status, self-efficacy, social functioning
Fortin *et al* (2021)^23^	Canada	294	Local organisation	HRQoL, functional status,^b^ self-efficacy
Gellis *et al* (2014)^24^	US	102	Local organisation	Functional status,^b^ social functioning^b^
Jäger *et al* (2017)^25^	Germany	273	Clinician	Social functioning, medication related^c^
Katon *et al* (2010)^26^	US	214	Local organisation	HRQoL
Köberlein-Neu *et al* (2016)^27^	Germany	162	Clinician	Functional status, social functioning, medication related^b,c^
Lin *et al* (2018)^28^	Taiwan	178	Clinician	HRQoL
Markle-Reid *et al* (2018)^29^	Canada	159	Local organisation	HRQoL,^b^ functional status,^b^ self-efficacy
Miklavcic *et al* (2020)^30^	Canada	132	Local organisation	Functional status,^b^ self-efficacy
Muth *et al* (2018)^31^	Germany	505	Clinician	HRQoL, functional status, medication related^c^
Rose *et al* (2018)^32^	Canada	470	Local organisation	HRQoL,^b^ self-efficacy, treatment adherence
Salisbury *et al* (2018)^33^ burden	UK	1546	Local organisation, higher health-services^d^	HRQoL,^b^ functional status, medication related,^c^ treatment
Siaw *et al* (2017)^34^	Singapore	411	Local organisation	Functional status
Stewart *et al* (2021)^35^	Canada	163	Local organisation	HRQoL, functional status,^b^ self-efficacy
Vera *et al* (2010)^36^	Puerto Rico	179	Local organisation	Social functioning
Von Korff *et al* (2011)^37^	US	214	Clinician, local organisation^d^	HRQoL,^b^ functional status
Webel *et al* (2018)^38^	US	179	Local organisation	HRQoL,^b^ functional status,^b^ self-efficacy,^b^ social functioning^b^
Zechmann *et al* (2020)^39^	Switzerland	336	Clinician	HRQoL, medication related^c^

a *Three levels have been identified within system-level interventions: individual clinician (for example, structured training regarding medication management); local healthcare provider (for example, multidisciplinary case conferences as part of collaborative care for patients with long-term conditions); higher health services (for example, multilocation collaborative care intervention). Comparator was usual care in all studies.*

b *Primary outcome measured within identified domain.*

c *Drugs per person, medication adherence, drug-related problems, and medication appropriateness are considered medication-related outcomes.*

d *Study deemed to cover more than one category regarding level of intervention. HRQoL = health-related quality of life.*

### Assessment of treatment burden outcome

Table 2 highlights the heterogeneity of outcome measures considered, and whether they were primary or secondary outcomes. Notably, just one study^33^ measured treatment burden directly, using the MTBQ; this was the largest study and was considered to be of high quality, with a complex, multicentre collaborative care intervention; however, it found no statistically significant improvement in treatment burden in patients receiving the interventions.

**Table 2.** Domains relating to treatment burden considered as outcomes among the included studies

**Study**	**Domains (origin) related to treatment burden covered by outcome measures**									
	**HRQoL (qualitative literature review^16^)**	**Functional status (MTBQ)**	**Self-efficacy (MTBQ)**	**Social functioning (PETS measure)**	**Medication management (PETS measure, MTBQ)**				**Treatment adherence (MTBQ)**	**Overall treatment burden**
					**Drugs per person, *n***	**Medication adherence**	**Drug-related problems**	**Medication appropriateness**		
Coventry *et al* (2015)^22^	X↔	X↔	X↔	X↔						
Fortin *et al* (2021)^23^	X↔	O↑	X↔							
Gellis *et al* (2014)^24^		O↑		O↔						
Jäger *et al* (2017)^25^				X↔		X↔				
Katon *et al* (2010)^26^	X↑									
Köberlein-Neu *et al* (2016)^27^		X↔		X↔			X↑	O↑		
Lin *et al* (2018)^28^	X↑									
Markle-Reid *et al* (2018)^29^	O↑	O↑	X↔							
Miklavcic *et al* (2020)^30^		O↔	X↔							
Muth *et al* (2018)^31^	X↔	X↔				X↔		O↔		
Rose *et al* (2018)^32^	O↔		X↔						X↔	
Salisbury *et al* (2018)^33^	O↔	X↔			X↔	X↔				X↔
Siaw *et al* (2017)^34^		X↑								
Stewart *et al* (2021)^35^	X↔	O↔	X↔							
Vera *et al* (2010)^36^				X↑						
Von Korff *et al* (2011)^37^	O↑	X↑								
Webel *et al* (2018)^38^	O↔	O↑	O↔	O↔						
Zechmann *et al* (2020)^39^	X↔				O↔					

*O = primary outcome. X = secondary outcome. ↑ = statistically significant improvement in outcome following intervention (*P≤*0.05). ↔ = no statistically significant effect of intervention on outcome measure (*P>*0.05). HRQoL = health-related quality of life. MTBQ = Multimorbidity Treatment Burden Questionnaire. PETS = Patient Experience of Treatment Survey.*

Outcome measurements most commonly covered the domains relating to impact on HRQoL and functional status, although impact on self-efficacy and social functioning were also covered in a number of studies. Five studies^25^^,^^26^^,^^31^^,^^33^^,^^39^ focused on outcomes related to management. Outcome measurement tools were very heterogeneous across studies under each domain, for example, across the 12 studies^22^^,^^23^^,^^26^^,^^28^^,^^29^^,^^31^^–^33^,^^35^^,^^37^^–^39 with outcomes relevant to the domain covering impact on HRQoL, seven different measurement tools were used to assess the outcome. Only some studies measured a primary outcome related to treatment burden domains, but most measured more than one outcome that could be placed within treatment burden domains.

### Evidence map

**Figure 2.**
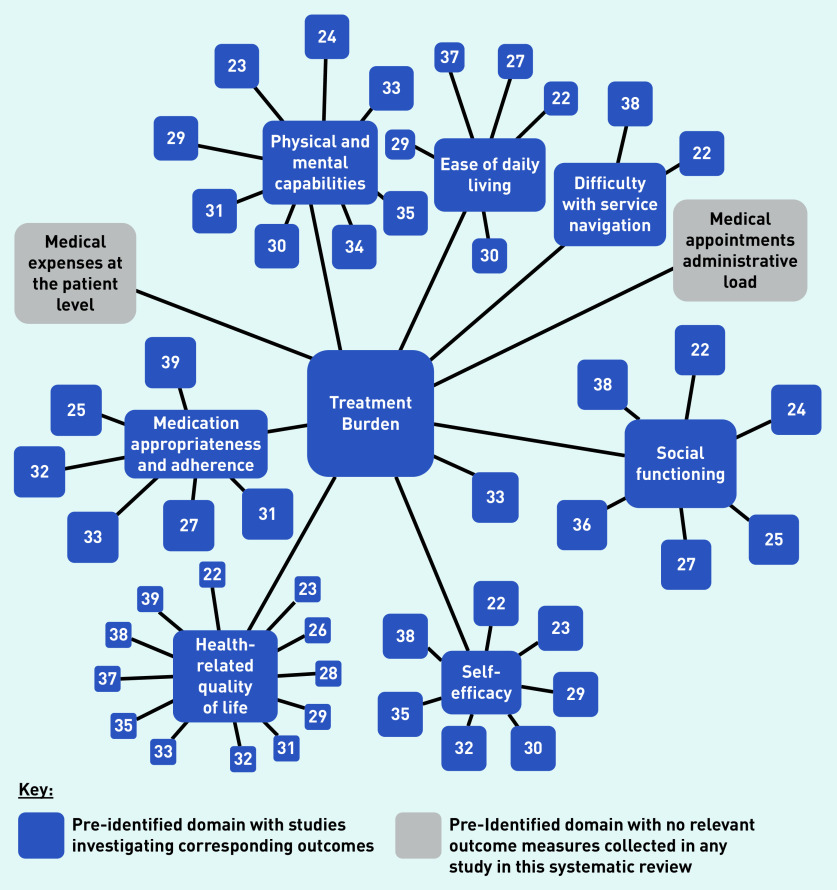
*Evidence map displaying study treatment burden domains identified a priori, which measure an outcome relevant to the displayed domain.^a^* *^a^For the purposes of this evidence map, functional status was considered to include ease of daily living, physical and mental capabilities, and difficulty with service navigation.*

The evidence map revealed that treatment burden domains considering medical costs and administrative task load at the patient level were not investigated in any of the 18 studies identified in the review presented here (Figure 2).

### Quality assessment

Most studies were considered to have some concern for risk of bias using the Cochrane RoB2 tool (Supplementary Table S3). Intervention adherence was mentioned in 11 of 18 studies^22^^–^26^,^^29^^,^^30^^,^^32^^,^^33^^,^^35^^,^^38^ and intervention dose was considered suboptimal in most studies,^22^^,^^23^^,^^25^^,^^30^^,^^32^^,^^33^^,^^35^ as implementation of system-level interventions proved complex, potentially reducing intervention effectiveness. Additionally, bias was a possibility in almost all studies as a result of the difficulty in blinding study assessors and participants. Application of the GRADE approach resulted in low degree of confidence in the evidence in this review (Supplementary Table S4).

## DISCUSSION

### Summary

This systematic review aimed to identify and synthesise the findings of studies that implemented system-level interventions and measured outcomes relating to treatment burden domains. A total of 18 RCTs were included, with interventions ranging from medication management changes at practitioner level to national collaborative care approaches. Seven predefined treatment burden domains were covered by outcomes measured in the included studies. Studies were heterogeneous in terms of interventions and outcome measures, and there were some concerns about risk of bias for most studies.

There was some evidence of intervention effect at local organisation level, particularly for those interventions involving collaborative care with significant patient involvement, such as through individualised management plans. The impact was particularly evident in the domains of HRQoL and functional status; however, use of the GRADE approach suggested caution should be exerted regarding interpretation of the findings.

System-level interventions have great potential to reduce treatment burden for people with multimorbidity, but more evidence is needed to inform this process, including the development and adoption of standard definitions and treatment burden outcome measures.

### Strengths and limitations

This systematic review has several strengths. The protocol was registered with PROSPERO prior to commencement, and SWiM and PRISMA guidelines ensured a systematic approach and methodology was documented. The Cochrane RoB2 tool was used to critically appraise studies, and the GRADE approach was used to assess the confidence of the results.

The broad search terms used were, likely, sensitive enough to pick up key studies. In addition, a second reviewer independently checked the included studies against the inclusion criteria and performed study quality assessments using the Cochrane RoB2 tool; disagreements were resolved through a third reviewer. The use of domains allowed for the identification of studies with outcomes that were highly relevant to treatment burden, which would not have been considered eligible if the criteria were constrained to directly measured treatment burden. No previous systematic reviews explore intervention effects on treatment burden in this manner.

The review also had a number of limitations, however. Although published tools (PETS, MTBQ) and qualitative literature were used to predefine treatment burden domains for the searches, there is the possibility that potentially relevant domains and corresponding studies were excluded if they were not captured within these tools or the qualitative review. Some studies may have also been excluded if their outcome measures did not fit clearly into a domain.

Another limitation was a lack of a generally agreed definition of ‘systems level’; instead, principles of system-level changes were used to screen interventions for eligibility, potentially excluding studies with relevant interventions.^18^ Study heterogeneity precluded the use of meta-analysis.

Despite the transparent approach to grouping by outcome, the authors acknowledge that other groupings could have been selected. Given the heterogeneity of studies, it was not possible to use transformation to produce standardised metrics. Consequently, the influence of interventions on the domains identified in this review could not be synthesised fully and no overall effect measures or quantitative indications of an effect could be presented.

The possibility of a type-two error (concluding no effect on the treatment burden domain when, in fact, one existed) cannot be ruled out for several of the studies, as the outcome relevant to treatment burden was considered a secondary outcome. In addition, restricting studies to those published in English may also have resulted in the exclusion of studies conducted in different health systems, reducing the generalisability of this review.

### Comparison with existing literature

A recent Cochrane review^17^ explored interventions in primary care to improve a range of outcomes for patients with multiple LTCs; six of the 10 studies in that review considered local organisation-level interventions, involving case management and coordination. The results indicated that interventions were more effective when targeted at specific risk-factor management, but the overall conclusions of the study were limited because of the heterogeneity of interventions.^17^ The Cochrane review reflected issues — that were also identified in this systematic review — in conducting research with patients with multiple LTCs, where selection bias seems possible due to recruitment difficulties.^17^ As an example, patients with greater capacity are more likely to participate in a trial than those with less capacity, which is directly relevant to outcomes linked to treatment burden.^30^ This bias may, additionally, reduce scope for improvement from baseline, as patients participating tend to be those experiencing a lower treatment burden.^33^

The evidence map revealed the lack of research examining intervention effects on treatment burden domains concerning medical costs and administrative task load. These domains are important to consider, alongside others, in their contribution towards higher levels of treatment burden in patients with multiple LTCs.

### Implications for research and practice

The successful implementation of system-level change to reduce treatment burden in patients with multiple LTCs requires further steps before conclusions can be drawn about the nature of system-level interventions most likely to be successful at reducing treatment burden. It is highly likely that the way in which services are organised has a substantial impact on the experience and work of being a patient, but this is difficult to demonstrate when direct measurement of treatment burden is seldom undertaken.

The review presented here suggests it may be beneficial for measures of treatment burden to be more routinely included in research and practice, to facilitate derivation of a standard outcome set. As an example, treatment burden could be listed as a standard outcome measure investigating organisational-level interventions and multimorbidity.^40^ This requires care so a measurement tool is not, in itself, burdensome. Treatment burden is a complex concept and developing an accurate and practicable measurement tool has proved challenging.^41^ In practice, currently validated measures may be time consuming to use. A single-item measure has been explored for use in clinical practice to identify patients who benefit from minimising avoidable burden; however, it only demonstrated moderate accuracy in comparison with more-complete measures and needs further consideration, potentially as a screening tool.^42^ Further thought could be given to the recruitment of patients with multiple LTCs in research, as patients may be more likely to participate when experiencing lower treatment burden levels.^43^

Common themes arising in the risk-of-bias assessment included poor intervention fidelity, inadequate follow-up duration, and a lack of blinding of outcome assessors. Future trials could carefully consider these issues in study design to reduce the risk of bias.

A broader understanding of health systems beyond the biomedical sphere may be generated by integrating complexity science in multimorbidity research to identify potential system-level improvements.^43^ System-level research may also benefit from the development of a standard definition for each type of care intervention; this might allow for greater comparison between studies and the impact of such interventions on treatment burden.

Further research on treatment burden domains not addressed by the studies identified in this review could help to give a broader understanding of treatment burden. All studies included in this review were based in a primary care setting; this may reflect where the majority of people with multiple LTCs are managed, but may also indicate that there is opportunity for further research on multimorbidity and treatment burden research in secondary care.

Patient-level approaches of care in integrated systems are likely to be helpful in reducing treatment burden for people with multiple LTCs. The movement of healthcare systems towards digital care may, however, exacerbate treatment burden as care responsibility is increasingly placed with patients, and may further disadvantage some population groups, thereby widening health inequalities.^44^
